# Supplementary material for: A UMLS-based spell checker for natural language processing in vaccine safety
Source: BMC Med Inform Decis Mak. 2007 Feb 12;7:3. doi: 10.1186/1472-6947-7-3 (PMC1805499; doi:10.1186/1472-6947-7-3)
Supplement: Additional file 13 — RAP application directory. Sets up directory for RAP (RDF application for PHP) source code [file 1472-6947-7-3-S13.gz › rap/doc/phpdoc/elementindex.html]

xml version="1.0" encoding="iso-8859-1"?


## Full index

### Package indexes

- model
- dataset
- infModel
- resModel
- ontModel
- syntax
- rdql
- utility

  

a
b
c
d
e
f
g
h
i
k
l
m
n
o
p
q
r
s
t
u
v
w
\_

a

top

$anon
:   N3Serializer::$anon in N3Serializer.php

add
:   MemModel::add() in MemModel.php

    Adds a new triple to the MemModel without checking if the statement is already in the MemModel.

add
:   InfModel::add() in InfModel.php

    Adds a new triple to the Model without checking if the statement is already in the Model.

add
:   InfModelB::add() in InfModelB.php

    Adds a new triple to the Model without checking, if the statement is already in the Model. So if you want a duplicate free Model use the addWithoutDuplicates() function (which is slower then add())

add
:   InfModelF::add() in InfModelF.php

    Adds a new triple to the MemModel without checking if the statement is already in the MemModel.

add
:   DbModel::add() in DbModel.php

    Add a new triple to this DbModel.

add
:   ResModel::add() in ResModel.php

    Adds a new triple to the Model without checking if the statement is already in the Model.

add
:   ResContainer::add() in ResContainer.php

    Add a new value to a container.

add
:   ResList::add() in ResList.php

    Add the given value to the end of the list.

addAll
:   DatasetDb::addAll() in DatasetDb.php

    Add all named graphs of the other dataset to this dataset

addAll
:   DatasetMem::addAll() in DatasetMem.php

    Add all named graphs of the other dataset to this dataset

addAtIndex
:   ResSeq::addAtIndex() in ResSeq.php

    Insert a new member into the sequence at the specified position.

addComment
:   OntResource::addComment() in OntResource.php

    Add the given comment to this resource.

addDomain
:   OntProperty::addDomain() in OntProperty.php

    Add a resource representing the domain of this property.

addIsDefinedBy
:   OntResource::addIsDefinedBy() in OntResource.php

    Add a resource that is declared to provide a definition of this resource.

addLabelProperty
:   OntResource::addLabelProperty() in OntResource.php

    Add the given Label to this resource

addModel
:   ResModel::addModel() in ResModel.php

    Adds another model to this MemModel.

addModel
:   InfModelF::addModel() in InfModelF.php

    Adds another model to this MemModel.

addModel
:   DbModel::addModel() in DbModel.php

    Add the given model to this DbModel.

addModel
:   MemModel::addModel() in MemModel.php

    Adds another model to this MemModel.

addNamedGraph
:   DatasetDb::addNamedGraph() in DatasetDb.php

    Adds a NamedGraph to the set.

addNamedGraph
:   DatasetMem::addNamedGraph() in DatasetMem.php

    Adds a NamedGraph to the set. Will replace a NamedGraph with the same name that is already in the set.

addNamespace
:   DbModel::addNamespace() in DbModel.php

    Adds a namespace and prefix to the model.

addNamespace
:   ResModel::addNamespace() in ResModel.php

    Adds a namespace and prefix to the model.

addNamespace
:   MemModel::addNamespace() in MemModel.php

    Adds a namespace and prefix to the model.

addNamespacePrefix
:   RdfSerializer::addNamespacePrefix() in RdfSerializer.php

    Adds a new prefix/namespace combination.

addNSPrefix
:   N3Serializer::addNSPrefix() in N3Serializer.php

    Adds a new namespace prefix to use.

addParsedNamespaces
:   MemModel::addParsedNamespaces() in MemModel.php

    Adds the namespaces to the model. This method is called by the parser. !!!! addParsedNamespaces() not overwrites manual added namespaces in the model !!!!

addParsedNamespaces
:   ResModel::addParsedNamespaces() in ResModel.php

    Adds the namespaces to the model. This method is called by the parser. !!!! addParsedNamespaces() not overwrites manual added namespaces in the model !!!!

addParsedNamespaces
:   DbModel::addParsedNamespaces() in DbModel.php

    Adds the namespaces to the model. This method is called by the parser. !!!! addParsedNamespaces() not overwrites manual added namespaces in the model !!!!

addProperty
:   ResResource::addProperty() in ResResource.php

    Add a property to this resource.

addProperty
:   OntResource::addProperty() in OntResource.php

    Add a property to this resource.

addQuad
:   DatasetMem::addQuad() in DatasetMem.php

    Adds a quad to the Dataset. The argument must not contain any wildcards. If the quad is already present, nothing happens. A new named graph will automatically be created if necessary.

addQuad
:   DatasetDb::addQuad() in DatasetDb.php

    Adds a quad to the Dataset. The argument must not contain any wildcards. If the quad is already present, nothing happens. A new named graph will automatically be created if necessary.

addRange
:   OntProperty::addRange() in OntProperty.php

    Add a resource representing the range of this property.

addRDFType
:   OntResource::addRDFType() in OntResource.php

    Add the given class as one of the rdf:type's for this resource.

addSeeAlso
:   OntResource::addSeeAlso() in OntResource.php

    Add a resource that is declared to provided additional information about the definition of this resource.

addSubClass
:   OntClass::addSubClass() in OntClass.php

    Add a sub-class of this class.

addSubProperty
:   OntProperty::addSubProperty() in OntProperty.php

    Add a sub-property of this property.

addSuperClass
:   OntClass::addSuperClass() in OntClass.php

    Add a super-class of this class.

addSuperProperty
:   OntProperty::addSuperProperty() in OntProperty.php

    Add a super-property of this property.

addWithoutDuplicates
:   ResModel::addWithoutDuplicates() in ResModel.php

    Checks if a new statement is already in the Model and adds the statement, if it is not in the Model.

addWithoutDuplicates
:   DbModel::addWithoutDuplicates() in DbModel.php

    Alias for the method add()

addWithoutDuplicates
:   InfModelF::addWithoutDuplicates() in InfModelF.php

    Checks if a new statement is already in the MemModel and adds the statement, if it is not in the MemModel.

addWithoutDuplicates
:   MemModel::addWithoutDuplicates() in MemModel.php

    Checks if a new statement is already in the MemModel and adds the statement, if it is not in the MemModel.

ALL\_DIFFERENT
:   OWLVocabulary::ALL\_DIFFERENT() in OWLVocabulary.php

ALL\_VALUES\_FROM
:   OWLVocabulary::ALL\_VALUES\_FROM() in OWLVocabulary.php

ANNOTATION\_PROPERTY
:   OWLVocabulary::ANNOTATION\_PROPERTY() in OWLVocabulary.php

append
:   ResList::append() in ResList.php

    Answer a new list that is formed by adding each element of

asClass
:   OntResource::asClass() in OntResource.php

    Answer a view of this resource as a class

asIndividual
:   OntResource::asIndividual() in OntResource.php

    Answer a view of this resource as an Individual

asOntProperty
:   OntResource::asOntProperty() in OntResource.php

    Answer a view of this resource as a property

b

top

$bNode
:   N3Parser::$bNode in N3Parser.php

BACKWARD\_COMPATIBLE\_WITH
:   OWLVocabulary::BACKWARD\_COMPATIBLE\_WITH() in OWLVocabulary.php

BlankNode
:   BlankNode::BlankNode() in Blanknode.php

    Constructor You can supply a label or You supply a model and a unique ID is gernerated

BlankNode
:   BlankNode in Blanknode.php

    An RDF blank node.

Blanknode.php
:   Blanknode.php in Blanknode.php

c

top

CARDINALITY
:   OWLVocabulary::CARDINALITY() in OWLVocabulary.php

checkEntailment
:   InfRule::checkEntailment() in InfRule.php

    Checks, if this rule could entail a statement that matches

checkTrigger
:   InfRule::checkTrigger() in InfRule.php

    Checks, if the statement satisfies the trigger

clear
:   DatasetMem::clear() in DatasetMem.php

    Deletes all NamedGraphs from the set.

clear
:   DatasetDb::clear() in DatasetDb.php

    Deletes all NamedGraphs from the set.

close
:   MemModel::close() in MemModel.php

    Close the MemModel and free up resources held.

close
:   DbModel::close() in DbModel.php

    Close this DbModel

close
:   DbStore::close() in DbStore.php

    Close the DbStore.

COMMENT
:   OWLVocabulary::COMMENT() in OWLVocabulary.php

    Answer the predicate that denotes comment annotation on an ontology element.

COMMENT
:   RdfsVocabulary::COMMENT() in RdfsVocabulary.php

    Answer the predicate that denotes comment annotation on an ontology element.

compare
:   Statement::compare() in Statement.php

    Compares two statements and returns integer less than, equal to, or greater than zero.

COMPLEMENT\_OF
:   OWLVocabulary::COMPLEMENT\_OF() in OWLVocabulary.php

concatenate
:   ResList::concatenate() in ResList.php

    Change the tail of this list to point to the given list, so that this list becomes the list of the concatenation of the elements of both lists. This is a side-effecting operation on this list; for a non side-effecting alternative, see append.

configSortModel
:   RdfSerializer::configSortModel() in RdfSerializer.php

    Serializer congiguration: Sort Model Flag if the serializer should sort the model by subject before serializing.

configUseAttributes
:   RdfSerializer::configUseAttributes() in RdfSerializer.php

    Serializer congiguration: Use Attributes Flag if the serializer should serialize triples as XML attributes where possible.

configUseEntities
:   RdfSerializer::configUseEntities() in RdfSerializer.php

    Serializer congiguration: Use Entities Flag if the serializer should use entities for URIs.

configUseQnames
:   RdfSerializer::configUseQnames() in RdfSerializer.php

    Serializer congiguration: Use Qnames Flag if the serializer should use qualified names for RDF reserved words.

configUseXmlDeclaration
:   RdfSerializer::configUseXmlDeclaration() in RdfSerializer.php

    Serializer congiguration: Use XML Declaration Flag if the serializer should start documents with the xml declaration <?xml version="1.0" encoding="UTF-8" ?>.

cons
:   ResList::cons() in ResList.php

    Return a reference to a new list cell whose head is value and whose tail is this list.

contains
:   ResModel::contains() in ResModel.php

    Tests if the Model contains the given statement.

contains
:   MemModel::contains() in MemModel.php

    Tests if the MemModel contains the given triple.

contains
:   InfModelB::contains() in InfModelB.php

    Tests if the Model contains the given triple.

contains
:   ResList::contains() in ResList.php

    Answer true if the given node appears as the value of a value of any of the cells of this list.

contains
:   DbModel::contains() in DbModel.php

    Check if the DbModel contains the given statement

contains
:   ResContainer::contains() in ResContainer.php

    Determine whether the container contains a value

containsAll
:   ResModel::containsAll() in ResModel.php

    Determine if all of the statements in a model are also contained in this model.

containsAll
:   DbModel::containsAll() in DbModel.php

    Determine if all of the statements in the given model are also contained in this DbModel.

containsAll
:   MemModel::containsAll() in MemModel.php

    Determine if all of the statements in a model are also contained in this MemModel.

containsAny
:   MemModel::containsAny() in MemModel.php

    Determine if any of the statements in a model are also contained in this MemModel.

containsAny
:   ResModel::containsAny() in ResModel.php

    Determine if any of the statements in a model are also contained in this model.

containsAny
:   DbModel::containsAny() in DbModel.php

    Determine if any of the statements in the given model are also contained in this DbModel.

containsNamedGraph
:   DatasetDb::containsNamedGraph() in DatasetDb.php

    Tells wether the Dataset contains a NamedGraph.

containsNamedGraph
:   DatasetMem::containsNamedGraph() in DatasetMem.php

    Tells wether the Dataset contains a NamedGraph.

containsQuad
:   DatasetMem::containsQuad() in DatasetMem.php

    Tells wether the Dataset contains a quad or quads matching a pattern.

containsQuad
:   DatasetDb::containsQuad() in DatasetDb.php

    Tells wether the Dataset contains a quad or quads matching a pattern.

containsResource
:   ResModel::containsResource() in ResModel.php

    Determine if the node (ResResource / ResLiteral) $node appears in any statement of this model.

copy
:   ResList::copy() in ResList.php

    Answer a list that contains all of the elements of this list in the same order, but is a duplicate copy in the underlying model.

countGraphs
:   DatasetMem::countGraphs() in DatasetMem.php

    Returns the number of NamedGraphs in the set. Empty graphs are counted.

countGraphs
:   DatasetDb::countGraphs() in DatasetDb.php

    Returns the number of NamedGraphs in the set. Empty graphs are counted.

countQuads
:   DatasetDb::countQuads() in DatasetDb.php

    Counts the Quads in the RDF dataset. Identical Triples in different NamedGraphs are counted individually.

countQuads
:   DatasetMem::countQuads() in DatasetMem.php

    Counts the Quads in the RDF dataset. Identical Triples in different NamedGraphs are counted individually.

countResults
:   RdqlResultIterator::countResults() in RdqlResultIterator.php

    Returns the number of results.

createAlt
:   ResModel::createAlt() in ResModel.php

    Create a new RDF Container from type rdf:Alt This method may return an existing container with the correct URI and model, or it may construct a fresh one, as it sees fit.

createBag
:   ResModel::createBag() in ResModel.php

    Create a new RDF Container from type rdf:Bag This method may return an existing container with the correct URI and model, or it may construct a fresh one, as it sees fit.

createGraph
:   DatasetDb::createGraph() in DatasetDb.php

    Creates a new NamedGraph and adds it to the set. An existing graph with the same name will be replaced. But the old namedGraph remains in the database.

createGraph
:   DatasetMem::createGraph() in DatasetMem.php

    Creates a new NamedGraph and adds it to the set. An existing

createIndividual
:   OntModel::createIndividual() in OntModel.php

    Answer a resource that represents an Individual node in this model.

createInstance
:   OntClass::createInstance() in OntClass.php

    Answer a resource that represents an instance of this OntClass and Individual node in this model.

createList
:   ResModel::createList() in ResModel.php

    Create a new RDF Collection from type rdf:List This method may return an existing container with the correct URI and model, or it may construct a fresh one, as it sees fit.

createLiteral
:   ResModel::createLiteral() in ResModel.php

    Create an untyped literal from a String value with a specified language.

createOntClass
:   OntModel::createOntClass() in OntModel.php

    Answer a resource that represents a class description node in this model.

createOntProperty
:   OntModel::createOntProperty() in OntModel.php

    Answer a resource that represents an OntProperty node in this model.

createOrd
:   RDFUtil::createOrd() in RdfUtil.php

    Creates ordinal RDF resource out of an integer.

createProperty
:   ResModel::createProperty() in ResModel.php

    Create a new Property associated with this model.

createResource
:   ResModel::createResource() in ResModel.php

    Create a new resource associated with this model.

createSeq
:   ResModel::createSeq() in ResModel.php

    Create a new RDF Container from type rdf:Seq This method may return an existing container with the correct URI and model, or it may construct a fresh one, as it sees fit.

createTables
:   DbStore::createTables() in DbStore.php

    Create tables and indexes for the given database type.

createTypedLiteral
:   ResModel::createTypedLiteral() in ResModel.php

    Create a literal from a String value with the $dtype Datatype An existing literal of the right value may be returned, or a fresh one created.

current
:   StatementIterator::current() in StatementIterator.php

    Returns the current statement.

current
:   FindIterator::current() in FindIterator.php

    Returns the current matching statement.

current
:   IteratorAllGraphsMem::current() in IteratorAllGraphsMem.php

    Returns the current item

current
:   IterFind::current() in IterFind.php

    Returns the current item

current
:   RdqlResultIterator::current() in RdqlResultIterator.php

    Returns the current result.

current
:   IteratorFindQuadsMem::current() in IteratorFindQuadsMem.php

    Returns the current item

current
:   IteratorFindQuadsDb::current() in IteratorFindQuadsDb.php

    Returns the current item

current
:   ResIterator::current() in ResIterator.php

    Returns the current item

current
:   IteratorAllGraphsDb::current() in IteratorAllGraphsDb.php

    Returns the current item

d

top

$DAML\_NS
:   N3Parser::$DAML\_NS in N3Parser.php

$debug
:   N3Parser::$debug in N3Parser.php

$debug
:   NTripleSerializer::$debug in NTripleSerializer.php

$debug
:   N3Serializer::$debug in N3Serializer.php

$done
:   N3Serializer::$done in N3Serializer.php

Dataset
:   Dataset in Dataset.php

    Dataset implementation.

DatasetDb
:   DatasetDb in DatasetDb.php

    Dataset implementation.

DatasetDb
:   DatasetDb::DatasetDb() in DatasetDb.php

    Constructor You can supply a Dataset name.

datasetExists
:   DbStore::datasetExists() in DbStore.php

    Check if the Dataset with the given $datasetName is already stored in the database

DatasetMem
:   DatasetMem in DatasetMem.php

    In-memory implementation of a RDF dataset.

DatasetMem
:   DatasetMem::DatasetMem() in DatasetMem.php

    Constructor You can supply a Dataset name.

DatasetDb.php
:   DatasetDb.php in DatasetDb.php

DatasetMem.php
:   DatasetMem.php in DatasetMem.php

Dataset.php
:   Dataset.php in Dataset.php

DATATYPE
:   OWLVocabulary::DATATYPE() in OWLVocabulary.php

DATATYPE\_PROPERTY
:   OWLVocabulary::DATATYPE\_PROPERTY() in OWLVocabulary.php

DATATYPE\_RESTRICTION
:   OWLVocabulary::DATATYPE\_RESTRICTION() in OWLVocabulary.php

DATA\_RANGE
:   OWLVocabulary::DATA\_RANGE() in OWLVocabulary.php

DbModel
:   DbModel::DbModel() in DbModel.php

    Constructor Do not call this directly.

DbModel
:   DbModel in DbModel.php

    This class provides methods for manipulating DbModels from DbStore.

DbStore
:   DbStore in DbStore.php

    DbStore is a persistent store of RDF data using relational database technology.

DbStore
:   DbStore::DbStore() in DbStore.php

    Constructor: Set the database connection with the given parameters.

delete
:   DbModel::delete() in DbModel.php

    Remove this DbModel from database and clean up.

DEPRECATED\_CLASS
:   OWLVocabulary::DEPRECATED\_CLASS() in OWLVocabulary.php

DEPRECATED\_PROPERTY
:   OWLVocabulary::DEPRECATED\_PROPERTY() in OWLVocabulary.php

DIFFERENT\_FROM
:   OWLVocabulary::DIFFERENT\_FROM() in OWLVocabulary.php

DISJOINT\_WITH
:   OWLVocabulary::DISJOINT\_WITH() in OWLVocabulary.php

DISTINCT\_MEMBERS
:   OWLVocabulary::DISTINCT\_MEMBERS() in OWLVocabulary.php

DOMAIN
:   RdfsVocabulary::DOMAIN() in RdfsVocabulary.php

    Answer the predicate that denotes the domain of a property.

DOMAIN
:   OWLVocabulary::DOMAIN() in OWLVocabulary.php

    Answer the predicate that denotes the domain of a property.

DbModel.php
:   DbModel.php in DbModel.php

DbStore.php
:   DbStore.php in DbStore.php

e

top

entail
:   InfRule::entail() in InfRule.php

    Returns a infered InfStatement by evaluating the statement with the entailment rule.

entailStatement
:   InfModelF::entailStatement() in InfModelF.php

    Entails a statement by recursively using the \_entailStatementRec method.

equals
:   Quad::equals() in Quad.php

    Checks, if two quads are equal.

equals
:   ResResource::equals() in ResResource.php

    Checks if the resource equals another resource.

equals
:   ResModel::equals() in ResModel.php

    Checks if two models are equal.

equals
:   Statement::equals() in Statement.php

    Checks if two statements are equal.

equals
:   MemModel::equals() in MemModel.php

    Checks if two models are equal.

equals
:   DbModel::equals() in DbModel.php

    Check if two models are equal.

equals
:   Literal::equals() in Literal.php

    Checks if ihe literal equals another literal.

equals
:   Resource::equals() in Resource.php

    Checks if the resource equals another resource.

equals
:   BlankNode::equals() in Blanknode.php

    Checks if two blank nodes are equal.

EQUIVALENT\_CLASS
:   OWLVocabulary::EQUIVALENT\_CLASS() in OWLVocabulary.php

EQUIVALENT\_PROPERTY
:   OWLVocabulary::EQUIVALENT\_PROPERTY() in OWLVocabulary.php

escapeValue
:   RDFUtil::escapeValue() in RdfUtil.php

    Escapes < > and &

f

top

find
:   ResModel::find() in ResModel.php

    General method to search for triples.

find
:   MemModel::find() in MemModel.php

    General method to search for triples.

find
:   InfModelB::find() in InfModelB.php

    General method to search for triples.

find
:   DbModel::find() in DbModel.php

    General method to search for triples in the DbModel.

findAsIterator
:   InfModelB::findAsIterator() in InfModelB.php

    Returns a FindIterator for traversing the MemModel.

findAsIterator
:   MemModel::findAsIterator() in MemModel.php

    Returns a FindIterator for traversing the MemModel.

findCount
:   DbModel::findCount() in DbModel.php

    Search for triples and return the number of matches.

findCount
:   MemModel::findCount() in MemModel.php

    Searches for triples and returns the number of matches.

findFirstMatchingStatement
:   InfModelB::findFirstMatchingStatement() in InfModelB.php

    Searches for triples and returns the first matching statement.

findFirstMatchingStatement
:   ResModel::findFirstMatchingStatement() in ResModel.php

    Searches for triples and returns the first matching statement.

findFirstMatchingStatement
:   DbModel::findFirstMatchingStatement() in DbModel.php

    Search for triples and return the first matching statement.

findFirstMatchingStatement
:   MemModel::findFirstMatchingStatement() in MemModel.php

    Searches for triples and returns the first matching statement.

findForward
:   Model::findForward() in Model.php

    Searches for triples using find() and tracks forward blank nodes until the final objects in the retrieved subgraphs are all named resources.

findInDefaultGraph
:   DatasetMem::findInDefaultGraph() in DatasetMem.php

    Finds Statements that match a pattern in the default Graph. The argument may contain wildcards.

findInDefaultGraph
:   DatasetDb::findInDefaultGraph() in DatasetDb.php

    Finds Statements that match a pattern in the default Graph. The argument may contain wildcards.

findInNamedGraphs
:   DatasetDb::findInNamedGraphs() in DatasetDb.php

    Finds Statements that match a quad pattern. The argument may contain wildcards.

findInNamedGraphs
:   DatasetMem::findInNamedGraphs() in DatasetMem.php

    Finds Statements that match a quad pattern. The argument may contain wildcards.

FindIterator
:   FindIterator in FindIterator.php

    Iterator for traversing statements matching a searchpattern.

FindIterator
:   FindIterator::FindIterator() in FindIterator.php

    Constructor

findRegex
:   MemModel::findRegex() in MemModel.php

    Method to search for triples using Perl-style regular expressions.

findRegex
:   DbModel::findRegex() in DbModel.php

    Method to search for triples using Perl-style regular expressions.

findRegex
:   InfModel::findRegex() in InfModel.php

    Method to search for triples using Perl-style regular expressions.

findVocabulary
:   InfModel::findVocabulary() in InfModel.php

    Returns all tripels of a certain vocabulary.

findVocabulary
:   MemModel::findVocabulary() in MemModel.php

    Returns all tripels of a certain vocabulary.

findVocabulary
:   DbModel::findVocabulary() in DbModel.php

    Return all tripels of a certain vocabulary.

FUNCTIONAL\_PROPERTY
:   OWLVocabulary::FUNCTIONAL\_PROPERTY() in OWLVocabulary.php

FindIterator.php
:   FindIterator.php in FindIterator.php

g

top

generateModel
:   N3Parser::generateModel() in N3Parser.php

    Generate a new MemModel from an URI or file.

generateModel
:   GRDDLParser::generateModel() in GRDDLParser.php

    generates a MemModel and creates the DomDocument.

generateModel
:   RdfParser::generateModel() in RdfParser.php

    Generates a new MemModel from a URI, a file or from memory.

get
:   ResList::get() in ResList.php

    Returns the value of the list element at the specified position or null.

getAssociatedModel
:   ResLiteral::getAssociatedModel() in ResLiteral.php

    Get the reference to the assocoated model.

getAssociatedModel
:   ResResource::getAssociatedModel() in ResResource.php

    Get the reference to the assocoated model.

getBaseMemModel
:   InfModelB::getBaseMemModel() in InfModelB.php

    Create a MemModel containing only the base triples (without inferred statements) of the current InfModelB

getBaseMemModel
:   InfModelF::getBaseMemModel() in InfModelF.php

    Create a MemModel containing only the base triples (without inferred statements) of the current InfModelF

getBaseURI
:   ResModel::getBaseURI() in ResModel.php

    Return current baseURI.

getBaseURI
:   Model::getBaseURI() in Model.php

    Return current baseURI.

getComment
:   OntResource::getComment() in OntResource.php

    Answer the comment string for this object. If there is more than one such resource, an arbitrary selection is made.

getContentInArray
:   ResList::getContentInArray() in ResList.php

    Get all values in the list as an array of ResResources

getCurrentPosition
:   StatementIterator::getCurrentPosition() in StatementIterator.php

    Returns the current position of the iterator.

getCurrentPosition
:   RdqlResultIterator::getCurrentPosition() in RdqlResultIterator.php

    Returns the current position of the iterator.

getDatasetDb
:   DbStore::getDatasetDb() in DbStore.php

    Create a new instance of DatasetDb with the given $datasetName and load the corresponding values from the database.

getDatasetMem
:   ModelFactory::getDatasetMem() in ModelFactory.php

    Returns a NamedGraphSetMem.

getDatasetName
:   DatasetDb::getDatasetName() in DatasetDb.php

    Return the Dataset name.

getDatasetName
:   DatasetMem::getDatasetName() in DatasetMem.php

    Return the Dataset name.

getDatatype
:   Literal::getDatatype() in Literal.php

    Returns the datatype of the literal.

getDbModel
:   ModelFactory::getDbModel() in ModelFactory.php

    Returns a new DbModel using the database connection supplied by $dbStore.

getDbStore
:   ModelFactory::getDbStore() in ModelFactory.php

    Returns a database connection with the given parameters.

getDefault
:   ResAlt::getDefault() in ResAlt.php

    Return the default value for this resource

getDefaultDbModel
:   ModelFactory::getDefaultDbModel() in ModelFactory.php

    Returns a DbModel with the database connection defined in constants.php.

getDefaultGraph
:   DatasetDb::getDefaultGraph() in DatasetDb.php

    Returns a reference to the defaultGraph

getDefaultGraph
:   DatasetMem::getDefaultGraph() in DatasetMem.php

    Returns a reference to the defaultGraph

getDefaultModel
:   ModelFactory::getDefaultModel() in ModelFactory.php

    Returns a MemModel.

getDomain
:   OntProperty::getDomain() in OntProperty.php

    Answer a OntClass that represents the domain class of this property.

getEntailment
:   InfRule::getEntailment() in InfRule.php

getGraphName
:   NamedGraphMem::getGraphName() in NamedGraphMem.php

    Return the graph name.

getGraphName
:   NamedGraphDb::getGraphName() in NamedGraphDb.php

    Return the graph name.

getGraphName
:   Quad::getGraphName() in Quad.php

    Return the graph name.

getHead
:   ResList::getHead() in ResList.php

    Get the value that is associated with the head of the list.

getID
:   BlankNode::getID() in Blanknode.php

    Returns the ID of the blank node.

getIndexType
:   MemModel::getIndexType() in MemModel.php

    Returns the indextype: -1 if there is no index, 0 if there are indices over S,P,O(separate),

    1. if there is an index over SPO, 2 if there is an index over SP and 3 if

    there is an index over SO.

getInfModelB
:   ModelFactory::getInfModelB() in ModelFactory.php

    Returns a InfModelB.

getInfModelF
:   ModelFactory::getInfModelF() in ModelFactory.php

    Returns a InfModelF.

getInstanceRdfType
:   OntResource::getInstanceRdfType() in OntResource.php

    returns the rdf:type, that this distinct resource is instance of.

getIsAnon
:   ResResource::getIsAnon() in ResResource.php

    Determine whether this resource is an anonymous resource

getIsDefinedBy
:   OntResource::getIsDefinedBy() in OntResource.php

    Answer a resource that is declared to provide a definition of this resource.

getLabel
:   Literal::getLabel() in Literal.php

    Returns the string value of the literal.

getLabel
:   BlankNode::getLabel() in Blanknode.php

    Returns the ID of the blank node.

getLabel
:   Resource::getLabel() in Resource.php

    Returns the label of the resource, which is the URI of the resource.

getLabelObject
:   Statement::getLabelObject() in Statement.php

    Reurns the URI, text or bNode identifier of the statements's object.

getLabelPredicate
:   Statement::getLabelPredicate() in Statement.php

    Returns the URI of the statements's predicate.

getLabelProperty
:   OntResource::getLabelProperty() in OntResource.php

    Answer the label ResLiteral for this object.

getLabelSubject
:   Statement::getLabelSubject() in Statement.php

    Returns the URI or bNode identifier of the statements's subject.

getLanguage
:   Literal::getLanguage() in Literal.php

    Returns the language of the literal.

getLocalName
:   Resource::getLocalName() in Resource.php

    Returns the local name of the resource.

getLocalName
:   RDFUtil::getLocalName() in RdfUtil.php

    Delivers the Localname (without the namespace prefix) out of the URI of a Resource.

getMember
:   ResSeq::getMember() in ResSeq.php

    Get the member at a given index

getMembers
:   ResContainer::getMembers() in ResContainer.php

    Get an array of all resources that are values of this container

getMemModel
:   ModelFactory::getMemModel() in ModelFactory.php

    Returns a MemModel.

getMemModel
:   InfModelB::getMemModel() in InfModelB.php

    Create a MemModel containing all the triples (including inferred statements) of the current InfModelB

getMemModel
:   InfModelF::getMemModel() in InfModelF.php

    Create a MemModel containing all the triples (including inferred statements) of the current InfModelF

getMemModel
:   DbModel::getMemModel() in DbModel.php

    Create a MemModel containing all the triples of the current DbModel

getMemModelByRDQL
:   Model::getMemModelByRDQL() in Model.php

    Perform an RDQL query on this Model. Should work with all types of models.

getModel
:   ResModel::getModel() in ResModel.php

    Returns a reference to the underlying model (Mem/DB/InfModel) that contains the statements

getModel
:   DbStore::getModel() in DbStore.php

    Create a new instance of DbModel with the given $modelURI and load the corresponding values of modelID and baseURI from the database.

getModifiedFind
:   InfRule::getModifiedFind() in InfRule.php

    Returns a find-query that matches statements, whose entailed statements would match the supplied find query.

getNamedGraph
:   DatasetMem::getNamedGraph() in DatasetMem.php

    Returns the NamedGraph with a specific name from the Dataset.

getNamedGraph
:   DatasetDb::getNamedGraph() in DatasetDb.php

    Returns the NamedGraph with a specific name from the Dataset.

getNamedGraphDb
:   DbStore::getNamedGraphDb() in DbStore.php

    Create a new instance of namedGraphDb with the given $modelURI and graphName and load the corresponding values of modelID and baseURI from the database.

getNamespace
:   RDFUtil::getNamespace() in RdfUtil.php

    Extracts the namespace prefix out of the URI of a Resource.

getNamespace
:   Resource::getNamespace() in Resource.php

    Returns the namespace of the resource. May return null.

getNewDatasetDb
:   DbStore::getNewDatasetDb() in DbStore.php

    Create a new instance of DatasetDb with the given $datasetName and insert the DatasetDb variables into the database.

getNewModel
:   DbStore::getNewModel() in DbStore.php

    Create a new instance of DbModel with the given $modelURI and insert the DbModel variables into the database.

getNewNamedGraphDb
:   DbStore::getNewNamedGraphDb() in DbStore.php

    Create a new instance of namedGraphDb with the given $modelURI and graphName and insert the DbModel variables into the database (not the graphName. This is only stored persistently, when added to dataset).

getObject
:   Quad::getObject() in Quad.php

    Return the object.

getObject
:   Statement::getObject() in Statement.php

    Returns the object of the triple.

getOntModel
:   Model::getOntModel() in Model.php

    Returns an OntModel with this model as baseModel.

getOntModel
:   ModelFactory::getOntModel() in ModelFactory.php

    Returns an OntModel.

getOrd
:   RDFUtil::getOrd() in RdfUtil.php

    Converts an ordinal RDF resource to an integer.

getParsedNamespaces
:   DbModel::getParsedNamespaces() in DbModel.php

    Returns the models namespaces.

getParsedNamespaces
:   MemModel::getParsedNamespaces() in MemModel.php

    Returns the models namespaces.

getParsedNamespaces
:   ResModel::getParsedNamespaces() in ResModel.php

    Returns the models namespaces.

getPovs
:   N3Parser::getPovs() in N3Parser.php

    Gets a list of triples with same subject e.g. :Gunnar :firstname "Gunnar" ; :lastname "Grimnes.

getPredicate
:   Statement::getPredicate() in Statement.php

    Returns the predicate of the triple.

getPredicate
:   Quad::getPredicate() in Quad.php

    Return the predicate.

getPrefix
:   RDFUtil::getPrefix() in RdfUtil.php

    Short Prefix for known Namespaces by given URI

getProperty
:   ResResource::getProperty() in ResResource.php

    Answer some statement (this, p, O) in the associated model.

getProperty
:   ResModel::getProperty() in ResModel.php

    Answer a statement find(s, p, null) with ResResources(ResLiterals) from this model.

getPropertyValue
:   OntResource::getPropertyValue() in OntResource.php

    Answer the value of a given RDF property for this resource as $returnType, or null if it doesn't have one. If there is more than one RDF statement with the given property for the current value, it is not defined which of the values will be returned.

getRange
:   OntProperty::getRange() in OntProperty.php

    Answer a OntClass that represents the range class of this property.

getRDFType
:   OntResource::getRDFType() in OntResource.php

    Answer the rdf:type (ie the class) of this resource.

getResModel
:   ModelFactory::getResModel() in ModelFactory.php

    Returns a ResModel.

getResmodel
:   Model::getResmodel() in Model.php

    Returns a ResModel with this model as baseModel.

getResultLabels
:   RdqlResultIterator::getResultLabels() in RdqlResultIterator.php

    Returns the labels of the result as array.

getSchema
:   InfModel::getSchema() in InfModel.php

    Returns a model, containing all Statements, having a Predicate, that is supported by the inference.

getSeeAlso
:   OntResource::getSeeAlso() in OntResource.php

    Answer a resource that provides additional information about this resource.

getStatement
:   Quad::getStatement() in Quad.php

    Return the statement(subject,predicate,object).

getStatementIterator
:   MemModel::getStatementIterator() in MemModel.php

    Returns a StatementIterator for traversing the MemModel.

getStatementIterator
:   InfModelB::getStatementIterator() in InfModelB.php

    Returns a StatementIterator for traversing the Model.

getSubClass
:   OntClass::getSubClass() in OntClass.php

    Answer a class that is the sub-class of this class.

getSubject
:   Statement::getSubject() in Statement.php

    Returns the subject of the triple.

getSubject
:   Quad::getSubject() in Quad.php

    Return the subject.

getSubProperty
:   OntProperty::getSubProperty() in OntProperty.php

    Answer a property that is the sub-property of this property.

getSuperClass
:   OntClass::getSuperClass() in OntClass.php

    Answer a class that is the super-class of this class.

getSuperProperty
:   OntProperty::getSuperProperty() in OntProperty.php

    Answer a property that is the super-property of this property.

getTail
:   ResList::getTail() in ResList.php

    Get the list that is the tail of this list.

getTrigger
:   InfRule::getTrigger() in InfRule.php

getURI
:   Resource::getURI() in Resource.php

    Returns the URI of the resource.

getVocabulary
:   OntResource::getVocabulary() in OntResource.php

    Answer a reference to the ontology language profile that governs the ontology model to which this ontology resource is attached.

GRDDLParser
:   GRDDLParser in GRDDLParser.php

    A GRDDLParser.

guessName
:   RDFUtil::guessName() in RdfUtil.php

    Delivers the name out of the URI (without the namespace prefix).

guessNamespace
:   RDFUtil::guessNamespace() in RdfUtil.php

    Extracts the namespace prefix out of a URI.

guessPrefix
:   RDFUtil::guessPrefix() in RdfUtil.php

    Short Prefix for known and/or parsed Namespaces by given URI and Model Uses $default\_prefixes defined in constants.php and getParsedNamespaces() Returns FALSE if no matching prefix is found

GRDDLParser.php
:   GRDDLParser.php in GRDDLParser.php

h

top

hasComment
:   OntResource::hasComment() in OntResource.php

    Answer true if this resource has the given comment.

hasDefaultGraph
:   DatasetDb::hasDefaultGraph() in DatasetDb.php

    Returns true, if an defaultGraph exists. False otherwise

hasDefaultGraph
:   DatasetMem::hasDefaultGraph() in DatasetMem.php

    Returns true, if an defaultGraph exists. False otherwise

hasDomain
:   OntProperty::hasDomain() in OntProperty.php

    Answer true if the given resource a class specifying the domain of this property.

hashCode
:   Statement::hashCode() in Statement.php

    Retruns the hash code of the triple.

hasLabelProperty
:   OntResource::hasLabelProperty() in OntResource.php

    Answer true if this resource has the given label.

hasNext
:   RdqlResultIterator::hasNext() in RdqlResultIterator.php

    Returns TRUE if there are more results.

hasNext
:   StatementIterator::hasNext() in StatementIterator.php

    Returns TRUE if there are more statements.

hasNext
:   FindIterator::hasNext() in FindIterator.php

    Returns TRUE if there are more matching statements.

hasPrevious
:   RdqlResultIterator::hasPrevious() in RdqlResultIterator.php

    Returns TRUE if the first result has not been reached.

hasPrevious
:   StatementIterator::hasPrevious() in StatementIterator.php

    Returns TRUE if the first statement has not been reached.

hasProperty
:   ResResource::hasProperty() in ResResource.php

    Determine whether this resource has any values for a given property.

hasRange
:   OntProperty::hasRange() in OntProperty.php

    Answer true if the given resource a class specifying the range of this property.

hasRDFType
:   OntResource::hasRDFType() in OntResource.php

    Answer true if this resource has the given rdf:type.

hasSeeAlso
:   OntResource::hasSeeAlso() in OntResource.php

    Answer true if this resource has the given resource as a source of additional information.

hasSubclass
:   OntClass::hasSubclass() in OntClass.php

    Answer true if the given class is a sub-class of this class.

hasSubProperty
:   OntProperty::hasSubProperty() in OntProperty.php

    Answer true if the given property is a sub-property of this property.

hasSuperClass
:   OntClass::hasSuperClass() in OntClass.php

    Answer true if the given class is a super-class of this class.

hasSuperProperty
:   OntProperty::hasSuperProperty() in OntProperty.php

    Answer true if the given property is a super-property of this property.

HAS\_VALUE
:   OWLVocabulary::HAS\_VALUE() in OWLVocabulary.php

i

top

IteratorAllGraphsDb.php
:   IteratorAllGraphsDb.php in IteratorAllGraphsDb.php

IteratorAllGraphsMem.php
:   IteratorAllGraphsMem.php in IteratorAllGraphsMem.php

IteratorFindQuadsDb.php
:   IteratorFindQuadsDb.php in IteratorFindQuadsDb.php

IteratorFindQuadsMem.php
:   IteratorFindQuadsMem.php in IteratorFindQuadsMem.php

IMPORTS
:   OWLVocabulary::IMPORTS() in OWLVocabulary.php

INCOMPATIBLE\_WITH
:   OWLVocabulary::INCOMPATIBLE\_WITH() in OWLVocabulary.php

index
:   MemModel::index() in MemModel.php

    Builds a search index for the statements in the MemModel.

indexOf
:   ResSeq::indexOf() in ResSeq.php

    Return the index of a given member of the sequence.

indexOf
:   ResList::indexOf() in ResList.php

    Get the Position of the first occurrence of the given value in the list, or -1 if the value is not in the list.

Individual
:   Individual::Individual() in Individual.php

    Constructor You can supply a uri

Individual
:   Individual in Individual.php

    Interface that encapsulates an individual in an ontology, sometimes referred to as a fact or assertion.

InfModel
:   InfModel in InfModel.php

    A InfModel Model extends a MemModel , by adding the ability to infer statements from known statements and RDFS/OWL-Schematas.

InfModel
:   InfModel::InfModel() in InfModel.php

    Constructor You can supply a base\_uri

InfModelB
:   InfModelB::InfModelB() in InfModelB.php

    Constructor You can supply a base\_uri

InfModelB
:   InfModelB in InfModelB.php

    A InfModelB extends the InfModel Class, with a backward chaining algorithm.

InfModelF
:   InfModelF::InfModelF() in InfModelF.php

    Constructor You can supply a base\_uri

InfModelF
:   InfModelF in InfModelF.php

    A InfModelF extends the InfModel Class, with a forward chaining algorithm.

InfModelB.php
:   InfModelB.php in InfModelB.php

InfModelF.php
:   InfModelF.php in InfModelF.php

InfModel.php
:   InfModel.php in InfModel.php

InfRule.php
:   InfRule.php in InfRule.php

InfStatement.php
:   InfStatement.php in InfStatement.php

InfRule
:   InfRule in InfRule.php

    This class represents a single rule in a RDFS inference model.

infRule
:   InfRule::infRule() in InfRule.php

    Constructor

InfStatement
:   InfStatement in InfStatement.php

    An RDF statement which was entailed by a inference rule.

intersect
:   MemModel::intersect() in MemModel.php

    Returns a new MemModel containing all the statements which are in both this MemModel and another.

intersect
:   DbModel::intersect() in DbModel.php

    Return a new MemModel containing all the statements which are in both this model and the given model.

intersect
:   ResModel::intersect() in ResModel.php

    Returns a new model containing all the statements which are in both this model and another.

INTERSECTION\_OF
:   OWLVocabulary::INTERSECTION\_OF() in OWLVocabulary.php

INVERSE\_FUNCTIONAL\_PROPERTY
:   OWLVocabulary::INVERSE\_FUNCTIONAL\_PROPERTY() in OWLVocabulary.php

INVERSE\_OF
:   OWLVocabulary::INVERSE\_OF() in OWLVocabulary.php

isAlt
:   ResContainer::isAlt() in ResContainer.php

    Returns true, if this resource is a container from type rdf:alt

isBag
:   ResContainer::isBag() in ResContainer.php

    Returns true, if this resource is a container from type rdf:bag

isDefinedBy
:   OntResource::isDefinedBy() in OntResource.php

    Answer true if this resource is defined by the given resource.

isEmpty
:   ResList::isEmpty() in ResList.php

    Returns true, if this list is empty

isEmpty
:   DbModel::isEmpty() in DbModel.php

    Check if this DbModel is empty

isEmpty
:   DatasetMem::isEmpty() in DatasetMem.php

    Tells wether the set contains any NamedGraphs.

isEmpty
:   MemModel::isEmpty() in MemModel.php

    Checks if MemModel is empty

isEmpty
:   DatasetDb::isEmpty() in DatasetDb.php

    Tells wether the set contains any NamedGraphs.

isEmpty
:   ResModel::isEmpty() in ResModel.php

    Checks if MemModel is empty

isIndexed
:   MemModel::isIndexed() in MemModel.php

    Returns true if there is an index, false if not.

isRDF
:   RDFUtil::isRDF() in RdfUtil.php

    Tests if the URI of a resource belongs to the RDF syntax/model namespace.

isSeq
:   ResContainer::isSeq() in ResContainer.php

    Returns true, if this resource is a container from type rdf:seq

isSetup
:   DbStore::isSetup() in DbStore.php

    Checks if tables are setup for RAP

isValid
:   ResList::isValid() in ResList.php

    Answer true if the list is well-formed, by checking that each node is correctly typed, and has a head and tail pointer from the correct vocabulary

IS\_DEFINED\_BY
:   OWLVocabulary::IS\_DEFINED\_BY() in OWLVocabulary.php

    Answer the predicate that denotes isDefinedBy annotation on an ontology element

IS\_DEFINED\_BY
:   RdfsVocabulary::IS\_DEFINED\_BY() in RdfsVocabulary.php

    Answer the predicate that denotes isDefinedBy annotation on an ontology element

IteratorAllGraphsDb
:   IteratorAllGraphsDb::IteratorAllGraphsDb() in IteratorAllGraphsDb.php

    Constructor.

IteratorAllGraphsDb
:   IteratorAllGraphsDb in IteratorAllGraphsDb.php

    Implementation of a Graph iterator.

IteratorAllGraphsMem
:   IteratorAllGraphsMem in IteratorAllGraphsMem.php

    Implementation of a Graph iterator.

IteratorAllGraphsMem
:   IteratorAllGraphsMem::IteratorAllGraphsMem() in IteratorAllGraphsMem.php

    Constructor.

IteratorFindQuadsDb
:   IteratorFindQuadsDb::IteratorFindQuadsDb() in IteratorFindQuadsDb.php

    Constructor.

IteratorFindQuadsDb
:   IteratorFindQuadsDb in IteratorFindQuadsDb.php

    Implementation of a quad iterator.

IteratorFindQuadsMem
:   IteratorFindQuadsMem in IteratorFindQuadsMem.php

    Implementation of a quad iterator.

IteratorFindQuadsMem
:   IteratorFindQuadsMem::IteratorFindQuadsMem() in IteratorFindQuadsMem.php

    Constructor.

iterFind
:   MemModel::iterFind() in MemModel.php

    Returns a FindIterator for traversing the MemModel.

IterFind
:   IterFind::IterFind() in IterFind.php

    Constructor.

IterFind
:   IterFind in IterFind.php

    Implementation of a find-iterator which delivers statements or quads.

Individual.php
:   Individual.php in Individual.php

IterFind.php
:   IterFind.php in IterFind.php

k

top

key
:   IterFind::key() in IterFind.php

    Returns the key of the current item

key
:   ResIterator::key() in ResIterator.php

    Returns the key of the current item

key
:   IteratorFindQuadsMem::key() in IteratorFindQuadsMem.php

    Returns the key of the current item

key
:   IteratorFindQuadsDb::key() in IteratorFindQuadsDb.php

    Returns the key of the current item

key
:   IteratorAllGraphsMem::key() in IteratorAllGraphsMem.php

    Returns the key of the current item

key
:   IteratorAllGraphsDb::key() in IteratorAllGraphsDb.php

    Returns the key of the current item

l

top

LABEL
:   OWLVocabulary::LABEL() in OWLVocabulary.php

    Answer the predicate that denotes label annotation on an ontology element

LABEL
:   RdfsVocabulary::LABEL() in RdfsVocabulary.php

    Answer the predicate that denotes label annotation on an ontology element

listClasses
:   OntModel::listClasses() in OntModel.php

    Answer an array that ranges over all of the various forms of class description resource in this model.

listComments
:   OntResource::listComments() in OntResource.php

    Answer an array of all of the comment literals for this resource.

listDomain
:   OntProperty::listDomain() in OntProperty.php

    Answer an array of all of the declared domain classes of this property.

listGraphNames
:   DatasetMem::listGraphNames() in DatasetMem.php

    Returns the names of the namedGraphs in this set as strings in an array.

listGraphNames
:   DatasetDb::listGraphNames() in DatasetDb.php

    Returns the names of the namedGraphs in this set as strings in an array.

listInstances
:   OntClass::listInstances() in OntClass.php

    Answer an ResIterator over the individuals in the model that have this class among their types.

listIsDefinedBy
:   OntResource::listIsDefinedBy() in OntResource.php

    Answer an array of all of the resources that are declared to define this resource.

listLabelProperties
:   OntResource::listLabelProperties() in OntResource.php

    Answer an array of all of the label literals for this resource, with the given language, if $language is set.

listModels
:   DbStore::listModels() in DbStore.php

    List all DbModels stored in the database.

listNamedGraphs
:   DatasetDb::listNamedGraphs() in DatasetDb.php

    Returns an iterator over all NamedGraphs in the set.

listNamedGraphs
:   DatasetMem::listNamedGraphs() in DatasetMem.php

    Returns an iterator over all NamedGraphs in the set.

listObjects
:   ResModel::listObjects() in ResModel.php

    Returns a ResIterator with all objects in a model.

listObjectsOfProperty
:   ResModel::listObjectsOfProperty() in ResModel.php

    Returns a ResIterator with all objects with a given property and property value.

listProperties
:   ResResource::listProperties() in ResResource.php

    List all the values with the property p as statements in an array.

listProperty
:   OntResource::listProperty() in OntResource.php

    Answer an array of values of a given RDF property for this resource as $returnType, or null if it doesn't have one.

listRange
:   OntProperty::listRange() in OntProperty.php

    Answer an array of all of the declared range classes of this property.

listRDFTypes
:   OntResource::listRDFTypes() in OntResource.php

    Answer an array of the RDF classes to which this resource belongs.

listSeeAlso
:   OntResource::listSeeAlso() in OntResource.php

    Answer an array of all of the resources that are declared to provide addition information about this resource.

listSubClasses
:   OntClass::listSubClasses() in OntClass.php

    Answer an array over the classes that are declared to be sub-classes of this class.

listSubjects
:   ResModel::listSubjects() in ResModel.php

    Returns a ResIterator with all subjects in a model.

listSubjectsWithProperty
:   ResModel::listSubjectsWithProperty() in ResModel.php

    Returns a ResIterator with all subjects with a given property and property value.

listSubProperties
:   OntProperty::listSubProperties() in OntProperty.php

    Answer an array of all the properties that are declared to be sub-properties of this property. Each element of the iterator will be an OntProperty.

listSuperClasses
:   OntClass::listSuperClasses() in OntClass.php

    Answer an array over the classes that are declared to be super-classes of this class.

listSuperProperties
:   OntProperty::listSuperProperties() in OntProperty.php

    Answer an array of all the properties that are declared to be super-properties of this property. Each element of the iterator will be an OntProperty.

Literal
:   Literal in Literal.php

    An RDF literal.

Literal
:   Literal::Literal() in Literal.php

    Constructor

load
:   Model::load() in Model.php

    Load a model from a file containing RDF, N3, N-Triples or a xhtml document containing RDF.

load
:   ResModel::load() in ResModel.php

    Load a model from a file containing RDF, N3 or N-Triples.

load
:   InfModelF::load() in InfModelF.php

    Load a model from a file containing RDF, N3 or N-Triples.

loadFromFile
:   Dataset::loadFromFile() in Dataset.php

    Load a Dataset from File

loadFromString
:   Dataset::loadFromString() in Dataset.php

    Load a Datset from a string

Literal.php
:   Literal.php in Literal.php

m

top

$model
:   RdfParser::$model in RdfParser.php

$model
:   N3Serializer::$model in N3Serializer.php

$model
:   NTripleSerializer::$model in NTripleSerializer.php

$m\_attributeStatements
:   RdfSerializer::$m\_attributeStatements in RdfSerializer.php

$m\_baseURI
:   RdfSerializer::$m\_baseURI in RdfSerializer.php

$m\_contentStatements
:   RdfSerializer::$m\_contentStatements in RdfSerializer.php

$m\_currentSubject
:   RdfSerializer::$m\_currentSubject in RdfSerializer.php

$m\_defaultNamespaces
:   RdfSerializer::$m\_defaultNamespaces in RdfSerializer.php

$m\_groupTypeStatement
:   RdfSerializer::$m\_groupTypeStatement in RdfSerializer.php

$m\_namespaces
:   RdfSerializer::$m\_namespaces in RdfSerializer.php

$m\_nextAutomaticPrefixIndex
:   RdfSerializer::$m\_nextAutomaticPrefixIndex in RdfSerializer.php

$m\_out
:   RdfSerializer::$m\_out in RdfSerializer.php

$m\_rdfAboutElementText
:   RdfSerializer::$m\_rdfAboutElementText in RdfSerializer.php

$m\_rdfIDElementText
:   RdfSerializer::$m\_rdfIDElementText in RdfSerializer.php

$m\_rdfResourceElementText
:   RdfSerializer::$m\_rdfResourceElementText in RdfSerializer.php

$m\_statements
:   RdfSerializer::$m\_statements in RdfSerializer.php

MAGIC\_STRING
:   MAGIC\_STRING in N3Serializer.php

MAX\_CARDINALITY
:   OWLVocabulary::MAX\_CARDINALITY() in OWLVocabulary.php

MemModel
:   MemModel::MemModel() in MemModel.php

    Constructor You can supply a base\_uri

MemModel
:   MemModel in MemModel.php

    A MemModel is an RDF Model, which is stored in the main memory.

MIN\_CARDINALITY
:   OWLVocabulary::MIN\_CARDINALITY() in OWLVocabulary.php

Model
:   Model in Model.php

    Abstract superclass of MemModel and DbModel. A model is a programming interface to an RDF graph.

Model
:   Model::Model() in Model.php

    Notice for people who are used to work with older versions of RAP.

modelExists
:   DbStore::modelExists() in DbStore.php

    Check if the DbModel with the given modelURI is already stored in the database

ModelFactory
:   ModelFactory in ModelFactory.php

    ModelFactory is a static class which provides methods for creating different types of RAP models. RAP models have to be created trough a ModelFactory instead of creating them directly with the 'new' operator because of RAP's dynamic code inclusion mechanism.

MemModel.php
:   MemModel.php in MemModel.php

ModelFactory.php
:   ModelFactory.php in ModelFactory.php

Model.php
:   Model.php in Model.php

moveFirst
:   StatementIterator::moveFirst() in StatementIterator.php

    Moves the pointer to the first statement.

moveFirst
:   RdqlResultIterator::moveFirst() in RdqlResultIterator.php

    Moves the pointer to the first result.

moveLast
:   StatementIterator::moveLast() in StatementIterator.php

    Moves the pointer to the last statement.

moveLast
:   RdqlResultIterator::moveLast() in RdqlResultIterator.php

    Moves the pointer to the last result.

moveTo
:   StatementIterator::moveTo() in StatementIterator.php

    Moves the pointer to a specific statement.

moveTo
:   RdqlResultIterator::moveTo() in RdqlResultIterator.php

    Moves the pointer to a specific result.

n

top

NamedGraphDb.php
:   NamedGraphDb.php in NamedGraphDb.php

NamedGraphMem.php
:   NamedGraphMem.php in NamedGraphMem.php

Node.php
:   Node.php in Node.php

N3Parser
:   N3Parser::N3Parser() in N3Parser.php

    Constructor

N3Parser
:   N3Parser in N3Parser.php

    PHP Notation3 Parser

N3Serializer
:   N3Serializer in N3Serializer.php

    An abstract object.

N3Serializer
:   N3Serializer::N3Serializer() in N3Serializer.php

    Constructor

NamedGraphDb
:   NamedGraphDb in NamedGraphDb.php

    Persistent NamedGraph implementation that extends a DbModel.

NamedGraphDb
:   NamedGraphDb::NamedGraphDb() in NamedGraphDb.php

    Constructor Do not call this directly.

NamedGraphMem
:   NamedGraphMem in NamedGraphMem.php

    NamedGraph implementation that extends a MemModel with a name.

NamedGraphMem
:   NamedGraphMem::NamedGraphMem() in NamedGraphMem.php

    Constructor You have to supply a graph name. You can supply a URI

NAMESPACE
:   RdfsVocabulary::NAMESPACE() in RdfsVocabulary.php

    Answer the string that is the namespace prefix for this vocabulary

NAMESPACE
:   OWLVocabulary::NAMESPACE() in OWLVocabulary.php

next
:   RdqlResultIterator::next() in RdqlResultIterator.php

    Returns the next result array.

next
:   StatementIterator::next() in StatementIterator.php

    Returns the next statement.

next
:   ResIterator::next() in ResIterator.php

    Moves Iterator to the next item in the list

next
:   IteratorAllGraphsDb::next() in IteratorAllGraphsDb.php

    Moves Iterator to the next item in the list

next
:   IteratorAllGraphsMem::next() in IteratorAllGraphsMem.php

    Moves Iterator to the next item in the list

next
:   FindIterator::next() in FindIterator.php

    Returns the next matching statement.

next
:   IteratorFindQuadsDb::next() in IteratorFindQuadsDb.php

    Moves Iterator to the next item in the list

next
:   IteratorFindQuadsMem::next() in IteratorFindQuadsMem.php

    Moves Iterator to the next item in the list

next
:   IterFind::next() in IterFind.php

    Moves Iterator to the next item in the list

Node
:   Node in Node.php

    An abstract RDF node.

NOTHING
:   OWLVocabulary::NOTHING() in OWLVocabulary.php

NTripleSerializer
:   NTripleSerializer::NTripleSerializer() in NTripleSerializer.php

    Constructor

NTripleSerializer
:   NTripleSerializer in NTripleSerializer.php

    PHP N-Triple Serializer

N3Parser.php
:   N3Parser.php in N3Parser.php

N3Serializer.php
:   N3Serializer.php in N3Serializer.php

NTripleSerializer.php
:   NTripleSerializer.php in NTripleSerializer.php

o

top

$object
:   FindIterator::$object in FindIterator.php

$OWL\_NS
:   N3Parser::$OWL\_NS in N3Parser.php

Object
:   Object in Object.php

    An abstract object.

object
:   Statement::object() in Statement.php

    Alias for getObject()

OBJECT\_CLASS
:   OWLVocabulary::OBJECT\_CLASS() in OWLVocabulary.php

OBJECT\_PROPERTY
:   OWLVocabulary::OBJECT\_PROPERTY() in OWLVocabulary.php

OBJECT\_RESTRICTION
:   OWLVocabulary::OBJECT\_RESTRICTION() in OWLVocabulary.php

ONE\_OF
:   OWLVocabulary::ONE\_OF() in OWLVocabulary.php

ONTCLASS
:   OWLVocabulary::ONTCLASS() in OWLVocabulary.php

    Answer the resource that represents the class 'class' in this vocabulary.

OntClass
:   OntClass in OntClass.php

    Class that represents an ontology node characterising a class description.

ONTCLASS
:   RdfsVocabulary::ONTCLASS() in RdfsVocabulary.php

    Answer the resource that represents the class 'class' in this vocabulary.

OntClass
:   OntClass::OntClass() in OntClass.php

    Constructor You can supply a uri

OntModel
:   OntModel in OntModel.php

    Enhanced view of the model that is known to contain ontology data, under a

OntModel
:   OntModel::OntModel() in OntModel.php

    Constructor.

OntClass.php
:   OntClass.php in OntClass.php

OntModelP.php
:   OntModelP.php in OntModelP.php

OntModel.php
:   OntModel.php in OntModel.php

OntProperty.php
:   OntProperty.php in OntProperty.php

OntResource.php
:   OntResource.php in OntResource.php

OntVocabulary.php
:   OntVocabulary.php in OntVocabulary.php

OWLVocabulary.php
:   OWLVocabulary.php in OWLVocabulary.php

ONTOLOGY
:   OWLVocabulary::ONTOLOGY() in OWLVocabulary.php

OntProperty
:   OntProperty::OntProperty() in OntProperty.php

    Constructor.

OntProperty
:   OntProperty in OntProperty.php

    Class encapsulating a property in an ontology.

OntResource
:   OntResource in OntResource.php

    Provides a common super-type for all of the abstractions in this ontology representation package.

OntResource
:   OntResource::OntResource() in OntResource.php

    Constructor You can supply a uri

OntVocabulary
:   OntVocabulary in OntVocabulary.php

    Base Class that encapsulates the elements of a general vocabulary

ON\_PROPERTY
:   OWLVocabulary::ON\_PROPERTY() in OWLVocabulary.php

OWLVocabulary
:   OWLVocabulary in OWLVocabulary.php

    OWL vocabulary items

Object.php
:   Object.php in Object.php

p

top

$parsedNamespaces
:   N3Parser::$parsedNamespaces in N3Parser.php

$parseError
:   N3Parser::$parseError in N3Parser.php

$predicate
:   FindIterator::$predicate in FindIterator.php

$prefixes
:   N3Serializer::$prefixes in N3Serializer.php

parse
:   N3Parser::parse() in N3Parser.php

    This parses a N3 string and prints out the triples

parse2model
:   N3Parser::parse2model() in N3Parser.php

    This parses a N3 string and returns a memmodel

parseFile
:   TriXParser::parseFile() in TriXParser.php

    Parse from a file

parseQuery
:   RdqlParser::parseQuery() in RdqlParser.php

    Parse the given RDQL query string and return an array with query variables and constraints.

parseString
:   TriXParser::parseString() in TriXParser.php

    Parse an XML string

predicate
:   Statement::predicate() in Statement.php

    Alias for getPredicate()

previous
:   RdqlResultIterator::previous() in RdqlResultIterator.php

    Returns the previous result.

previous
:   StatementIterator::previous() in StatementIterator.php

    Returns the previous statement.

PRIOR\_VERSION
:   OWLVocabulary::PRIOR\_VERSION() in OWLVocabulary.php

PROPERTY
:   OWLVocabulary::PROPERTY() in OWLVocabulary.php

putModel
:   DbStore::putModel() in DbStore.php

    Store a MemModel or another DbModel from a different DbStore in the database.

q

top

Quad.php
:   Quad.php in Quad.php

Quad
:   Quad::Quad() in Quad.php

    Constructor Creates a Quad from four Nodes.

Quad
:   Quad in Quad.php

    A Triple in a RDF dataset, consisting of four Jena Nodes: graphName, subject, predicate, and object.

queryModel
:   RdqlMemEngine::queryModel() in RdqlMemEngine.php

    Perform an RDQL Query on the given MemModel.

queryModel
:   RdqlDbEngine::queryModel() in RdqlDbEngine.php

    Perform an RDQL Query on the given DbModel.

r

top

$RDF\_NS
:   N3Parser::$RDF\_NS in N3Parser.php

$rdf\_parser
:   RdfParser::$rdf\_parser in RdfParser.php

$rdf\_qnames
:   RdfSerializer::$rdf\_qnames in RdfSerializer.php

$rdf\_qname\_prefix
:   RdfSerializer::$rdf\_qname\_prefix in RdfSerializer.php

$res
:   N3Serializer::$res in N3Serializer.php

$res
:   NTripleSerializer::$res in NTripleSerializer.php

$resourcetext
:   N3Serializer::$resourcetext in N3Serializer.php

$resourcetext\_taken
:   N3Serializer::$resourcetext\_taken in N3Serializer.php

Resource.php
:   Resource.php in Resource.php

RdfsVocabulary.php
:   RdfsVocabulary.php in RdfsVocabulary.php

RANGE
:   OWLVocabulary::RANGE() in OWLVocabulary.php

    Answer the predicate that denotes the domain of a property.

RANGE
:   RdfsVocabulary::RANGE() in RdfsVocabulary.php

    Answer the predicate that denotes the domain of a property.

RdfParser
:   RdfParser in RdfParser.php

    An RDF paser.

RdfSerializer
:   RdfSerializer in RdfSerializer.php

    An RDF seralizer.

RdfSerializer
:   RdfSerializer::RdfSerializer() in RdfSerializer.php

    Constructor

RdfsVocabulary
:   RdfsVocabulary in RdfsVocabulary.php

    RDFS vocabulary items

RDFUtil
:   RDFUtil in RdfUtil.php

    Useful utility methods.

RdqlDbEngine
:   RdqlDbEngine in RdqlDbEngine.php

    This class performs as RDQL query on a DbModel.

RdqlEngine
:   RdqlEngine in RdqlEngine.php

    Some general methods common for RdqlMemEngine and RdqlDbEngine

RdqlMemEngine
:   RdqlMemEngine in RdqlMemEngine.php

    This class performes as RDQL query on a MemModel.

RdqlParser
:   RdqlParser in RdqlParser.php

    This class contains methods for parsing an Rdql query string into PHP variables.

rdqlQuery
:   ResModel::rdqlQuery() in ResModel.php

    Perform an RDQL query on this MemModel.

rdqlQuery
:   DbModel::rdqlQuery() in DbModel.php

    Perform an RDQL query on this DbModel.

rdqlQuery
:   MemModel::rdqlQuery() in MemModel.php

    Perform an RDQL query on this MemModel.

rdqlQueryAsIterator
:   ResModel::rdqlQueryAsIterator() in ResModel.php

    Perform an RDQL query on this MemModel.

rdqlQueryAsIterator
:   MemModel::rdqlQueryAsIterator() in MemModel.php

    Perform an RDQL query on this MemModel.

rdqlQueryAsIterator
:   DbModel::rdqlQueryAsIterator() in DbModel.php

    Perform an RDQL query on this DBModel.

RdqlResultIterator
:   RdqlResultIterator in RdqlResultIterator.php

    Iterator for traversing Rdql results.

RdqlResultIterator
:   RdqlResultIterator::RdqlResultIterator() in RdqlResultIterator.php

    Constructor

RdqlDbEngine.php
:   RdqlDbEngine.php in RdqlDbEngine.php

RdqlEngine.php
:   RdqlEngine.php in RdqlEngine.php

RdqlMemEngine.php
:   RdqlMemEngine.php in RdqlMemEngine.php

RdqlParser.php
:   RdqlParser.php in RdqlParser.php

RdqlResultIterator.php
:   RdqlResultIterator.php in RdqlResultIterator.php

reify
:   MemModel::reify() in MemModel.php

    Reifies the MemModel.

reify
:   DbModel::reify() in DbModel.php

    Reify the DbModel.

reify
:   Statement::reify() in Statement.php

    Reifies a statement.

remove
:   ResModel::remove() in ResModel.php

    Removes the statement of ResResources(ResTriples) from the MemModel.

remove
:   ResList::remove() in ResList.php

    Remove the given value from this list.

remove
:   ResContainer::remove() in ResContainer.php

    Remove a value from the container.

remove
:   MemModel::remove() in MemModel.php

    Removes the triple from the MemModel.

remove
:   InfModelF::remove() in InfModelF.php

    Removes the triple from the MemModel.

remove
:   InfModelB::remove() in InfModelB.php

    Removes the triple from the MemModel.

remove
:   DbModel::remove() in DbModel.php

    Remove the given triple from this DbModel.

removeAll
:   ResResource::removeAll() in ResResource.php

    Delete all the statements with predicate p for this resource from its associated model.

removeAtIndex
:   ResSeq::removeAtIndex() in ResSeq.php

    Remove the member at the specified index.

removeComment
:   OntResource::removeComment() in OntResource.php

    Remove the statement that the given ResLiteral is a comment on this resource.

removeDefinedBy
:   OntResource::removeDefinedBy() in OntResource.php

    Remove the statement that this resource is defined by the given resource.

removeDomain
:   OntProperty::removeDomain() in OntProperty.php

    Remove the given class from the stated domain(s) of this property.

removeHead
:   ResList::removeHead() in ResList.php

    Remove the head of the list. The tail of the list remains in the model. Note that no changes are made to list cells that point to this list cell as their tail.

removeInfered
:   InfModelF::removeInfered() in InfModelF.php

    Removes all infered statements from the model but keeps the infernece rules.

removeLabelProperty
:   OntResource::removeLabelProperty() in OntResource.php

    Remove the statement that the given ResLiteral is a label on this resource.

removeList
:   ResList::removeList() in ResList.php

    Remove all of the components of this list from the model.

removeNamedGraph
:   DatasetMem::removeNamedGraph() in DatasetMem.php

    Removes a NamedGraph from the set. Nothing happens if no graph with that name is contained in the set.

removeNamedGraph
:   DatasetDb::removeNamedGraph() in DatasetDb.php

    Removes a NamedGraph from the set. Nothing happens if no graph with that name is contained in the set.

removeNamedGraphDb
:   DbStore::removeNamedGraphDb() in DbStore.php

    Removes the graph with all statements from the database.

removeNamespace
:   MemModel::removeNamespace() in MemModel.php

    removes a single namespace from the model

removeNamespace
:   DbModel::removeNamespace() in DbModel.php

    removes a single namespace from the model

removeNamespace
:   ResModel::removeNamespace() in ResModel.php

    removes a single namespace from the model

removeProperties
:   ResResource::removeProperties() in ResResource.php

    Delete all the properties for this resource from the associated model.

removeProperty
:   OntResource::removeProperty() in OntResource.php

    Remove the specific property-value pair from this resource.

removeQuad
:   DatasetDb::removeQuad() in DatasetDb.php

    Deletes a Quad from the RDF dataset.

removeQuad
:   DatasetMem::removeQuad() in DatasetMem.php

    Deletes a Quad from the RDF dataset.

removeRange
:   OntProperty::removeRange() in OntProperty.php

    Remove the given class from the stated range(es) of this property.

removeRDFType
:   OntResource::removeRDFType() in OntResource.php

    Remove the statement that this resource is of the given RDF type.

removeSeeAlso
:   OntResource::removeSeeAlso() in OntResource.php

    Remove the statement indicating the given resource as a source of additional information about this resource.

removeSubClass
:   OntClass::removeSubClass() in OntClass.php

    Remove the given class from the sub-classes of this class.

removeSubProperty
:   OntProperty::removeSubProperty() in OntProperty.php

    Remove the given property from the sub-properties of this property.

removeSuperClass
:   OntClass::removeSuperClass() in OntClass.php

    Remove the given class from the super-classes of this class.

removeSuperProperty
:   OntProperty::removeSuperProperty() in OntProperty.php

    Remove the given property from the super-properties of this property.

replace
:   InfModel::replace() in InfModel.php

    General method to replace nodes of a MemModel.

replace
:   MemModel::replace() in MemModel.php

    General method to replace nodes of a MemModel.

replace
:   ResList::replace() in ResList.php

    Replace the value at the i'th position in the list with the given value

replace
:   DbModel::replace() in DbModel.php

    General method to replace nodes of a DbModel.

ResAlt
:   ResAlt in ResAlt.php

    This interface defines methods for accessing RDF Alternative resources.

ResAlt
:   ResAlt::ResAlt() in ResAlt.php

    Constructor You can supply a URI

ResBag
:   ResBag in ResBag.php

    This interface defines methods for accessing RDF Bag resources.

ResBag
:   ResBag::ResBag() in ResBag.php

    Constructor You can supply a URI

ResContainer
:   ResContainer::ResContainer() in ResContainer.php

    Constructor You can supply a URI

ResContainer
:   ResContainer in ResContainer.php

    An RDF Container.

ResIterator
:   ResIterator::ResIterator() in ResIterator.php

    Constructor.

ResIterator
:   ResIterator in ResIterator.php

    Implementation of a resource iterator.

ResList
:   ResList::ResList() in ResList.php

    Constructor You can supply a URI

ResList
:   ResList in ResList.php

    Implementation of an rdf:Collection (rdf:List) Provides a convenience encapsulation for lists formed from chains of RDF statements arranged to form a head/tail cons-cell structure.

ResLiteral
:   ResLiteral in ResLiteral.php

    An RDF literal.

ResLiteral
:   ResLiteral::ResLiteral() in ResLiteral.php

    Constructor You have to supply a string.

ResModel
:   ResModel::ResModel() in ResModel.php

    Constructor You have to supply a memmodel/dbmodel/infmodel to save the statements.

ResModel
:   ResModel in ResModel.php

    A ResModel provides an resource centric view on an underlying RDF model.

ResAlt.php
:   ResAlt.php in ResAlt.php

ResBag.php
:   ResBag.php in ResBag.php

ResContainer.php
:   ResContainer.php in ResContainer.php

ResIterator.php
:   ResIterator.php in ResIterator.php

ResList.php
:   ResList.php in ResList.php

ResLiteral.php
:   ResLiteral.php in ResLiteral.php

ResModel.php
:   ResModel.php in ResModel.php

ResProperty.php
:   ResProperty.php in ResProperty.php

ResResource.php
:   ResResource.php in ResResource.php

ResSeq.php
:   ResSeq.php in ResSeq.php

Resource
:   Resource::Resource() in Resource.php

    Constructor Takes an URI or a namespace/localname combination

Resource
:   Resource in Resource.php

    An RDF resource.

ResProperty
:   ResProperty in ResProperty.php

    An RDF Property.

ResProperty
:   ResProperty::ResProperty() in ResProperty.php

    Constructor You can supply a URI

ResResource
:   ResResource in ResResource.php

    An RDF Resource.

ResResource
:   ResResource::ResResource() in ResResource.php

    Constructor You can supply a uri

ResSeq
:   ResSeq in ResSeq.php

    This interface defines methods for accessing RDF Sequence resources.

ResSeq
:   ResSeq::ResSeq() in ResSeq.php

    Constructor You can supply a URI

RESTRICTION
:   OWLVocabulary::RESTRICTION() in OWLVocabulary.php

rewind
:   IteratorAllGraphsMem::rewind() in IteratorAllGraphsMem.php

    Resets iterator list to start

rewind
:   IteratorFindQuadsMem::rewind() in IteratorFindQuadsMem.php

    Resets iterator list to start

rewind
:   IterFind::rewind() in IterFind.php

    Resets iterator list to start

rewind
:   IteratorAllGraphsDb::rewind() in IteratorAllGraphsDb.php

    Resets iterator list to start

rewind
:   IteratorFindQuadsDb::rewind() in IteratorFindQuadsDb.php

    Resets iterator list to start

rewind
:   ResIterator::rewind() in ResIterator.php

    Resets iterator list to start

RdfParser.php
:   RdfParser.php in RdfParser.php

RdfSerializer.php
:   RdfSerializer.php in RdfSerializer.php

RdfUtil.php
:   RdfUtil.php in RdfUtil.php

s

top

$sort\_model
:   RdfSerializer::$sort\_model in RdfSerializer.php

Statement.php
:   Statement.php in Statement.php

sameListAs
:   ResList::sameListAs() in ResList.php

    Answer true if this list has the same elements in the same order as the given list. Note that the standard equals test just tests for equality of two given list cells.

SAME\_AS
:   OWLVocabulary::SAME\_AS() in OWLVocabulary.php

SAME\_CLASS\_AS
:   OWLVocabulary::SAME\_CLASS\_AS() in OWLVocabulary.php

SAME\_INDIVIDUAL\_AS
:   OWLVocabulary::SAME\_INDIVIDUAL\_AS() in OWLVocabulary.php

SAME\_PROPERTY\_AS
:   OWLVocabulary::SAME\_PROPERTY\_AS() in OWLVocabulary.php

saveAs
:   NTripleSerializer::saveAs() in NTripleSerializer.php

    Serializes a model and saves it into a file.

saveAs
:   RdfSerializer::saveAs() in RdfSerializer.php

    Serializes a model and saves it into a file.

saveAs
:   ResModel::saveAs() in ResModel.php

    Saves the RDF,N3 or N-Triple serialization of the MemModel to a file.

saveAs
:   MemModel::saveAs() in MemModel.php

    Saves the RDF,N3 or N-Triple serialization of the MemModel to a file.

saveAs
:   InfModelB::saveAs() in InfModelB.php

    Saves the RDF,N3 or N-Triple serialization of the full InfModelB (including inferred triples) to a file.

saveAs
:   DbModel::saveAs() in DbModel.php

    Saves the RDF,N3 or N-Triple serialization of the DbModel to a file.

saveAs
:   N3Serializer::saveAs() in N3Serializer.php

    Serializes a model and saves it into a file.

SEE\_ALSO
:   RdfsVocabulary::SEE\_ALSO() in RdfsVocabulary.php

    Answer the predicate that denotes seeAlso annotation on an ontology element

SEE\_ALSO
:   OWLVocabulary::SEE\_ALSO() in OWLVocabulary.php

    Answer the predicate that denotes seeAlso annotation on an ontology element

serialize
:   N3Serializer::serialize() in N3Serializer.php

    Serializes a model to N3 syntax.

serialize
:   RdfSerializer::serialize() in RdfSerializer.php

    Serializes a model to RDF syntax.

serialize
:   NTripleSerializer::serialize() in NTripleSerializer.php

    Serializes a model to N Triple syntax.

serializeToFile
:   Dataset::serializeToFile() in Dataset.php

    Serialize the Dataset to File

serializeToFile
:   TriXSerializer::serializeToFile() in TriXSerializer.php

    Serialize the dataset to a TriX string and save in file

serializeToString
:   Dataset::serializeToString() in Dataset.php

    Serialize the Dataset to string

serializeToString
:   TriXSerializer::serializeToString() in TriXSerializer.php

    Serialize the dataset to a TriX string

set
:   ResSeq::set() in ResSeq.php

    Set the value at a given index in the sequence.

setAssociatedModel
:   ResLiteral::setAssociatedModel() in ResLiteral.php

    Sets the reference to the assocoated model.

setAssociatedModel
:   ResResource::setAssociatedModel() in ResResource.php

    Sets the reference to the assocoated model.

setBaseURI
:   ResModel::setBaseURI() in ResModel.php

    Set a base URI for the MemModel.

setBaseURI
:   MemModel::setBaseURI() in MemModel.php

    Set a base URI for the MemModel.

setBaseURI
:   DbModel::setBaseURI() in DbModel.php

    Set a base URI for the DbModel.

setComment
:   OntResource::setComment() in OntResource.php

    Assert that the given string is the comment on this resource.

setDatasetName
:   DatasetDb::setDatasetName() in DatasetDb.php

    Sets the Dataset name. Return true on success, false otherwise

setDatasetName
:   DatasetMem::setDatasetName() in DatasetMem.php

    Sets the Dataset name.

setDatatype
:   Literal::setDatatype() in Literal.php

    Sets the datatype of the literal.

setDefault
:   ResAlt::setDefault() in ResAlt.php

    Set the default value of this container.

setDefaultGraph
:   DatasetMem::setDefaultGraph() in DatasetMem.php

    Overwrites the existting default graph.

setDefaultGraph
:   DatasetDb::setDefaultGraph() in DatasetDb.php

    Overwrites the existting default graph.

setDomain
:   OntProperty::setDomain() in OntProperty.php

    Assert that the given resource represents the class of individuals that form the domain of this property. Any existing domain statements for this property are removed.

setEntailment
:   InfRule::setEntailment() in InfRule.php

    Sets the entailment of this rule The values can be NULL to match anything or be a node that has to be matched.

setFindLiteralWithLang
:   ResIterator::setFindLiteralWithLang() in ResIterator.php

    Sets that only Literals with the matching language should be returned

setFixBnodes
:   N3Parser::setFixBnodes() in N3Parser.php

    Sets, if BlankNode labels should be replaced by the generic label from the constants.php file

setGraphName
:   NamedGraphMem::setGraphName() in NamedGraphMem.php

    Sets the graph name.

setGraphName
:   NamedGraphDb::setGraphName() in NamedGraphDb.php

    Sets the graph name.

setGraphName
:   Quad::setGraphName() in Quad.php

    Sets the graph name.

setHead
:   ResList::setHead() in ResList.php

    Update the head of the list to have the given value, and return the previous value.

setInstanceRdfType
:   OntResource::setInstanceRdfType() in OntResource.php

    Sets the rdf:type, that this distinct resource is instance of.

setIsAnon
:   ResResource::setIsAnon() in ResResource.php

    Set whether this resource is an anonymous resource

setIsDefinedBy
:   OntResource::setIsDefinedBy() in OntResource.php

    Assert that the given resource provides a source of definitions about this resource.

setLabelProperty
:   OntResource::setLabelProperty() in OntResource.php

    Assert that the given string is the label on this resource.

setLanguage
:   Literal::setLanguage() in Literal.php

    Sets the language of the literal.

setPropertyValue
:   OntResource::setPropertyValue() in OntResource.php

    Set the value of the given property of this ontology resource to the given value.

setRange
:   OntProperty::setRange() in OntProperty.php

    Assert that the given resource represents the class of individuals that form the range of this property. Any existing range statements for this property are removed.

setRDFType
:   OntResource::setRDFType() in OntResource.php

    Set the RDF type (ie the class) for this resource, replacing any existing rdf:type property. Any existing statements for the RDF type will first be removed.

setSeeAlso
:   OntResource::setSeeAlso() in OntResource.php

    Add a resource that is declared to provided additional information about the definition of this resource

setSubClass
:   OntClass::setSubClass() in OntClass.php

    Assert that this class is super-class of the given class.

setSubProperty
:   OntProperty::setSubProperty() in OntProperty.php

    Assert that this property is super-property of the given property.

setSuperClass
:   OntClass::setSuperClass() in OntClass.php

    Assert that this class is sub-class of the given class.

setSuperProperty
:   OntProperty::setSuperProperty() in OntProperty.php

    Assert that this property is sub-property of the given property.

setTail
:   ResList::setTail() in ResList.php

    Update the list cell at the front of the list to have the given list as tail. The old tail is returned, and remains in the model.

setTrigger
:   InfRule::setTrigger() in InfRule.php

    Sets the trigger of this rule The values can be NULL to match anything or be a node that has to be matched.

setURI
:   ResResource::setURI() in ResResource.php

    Sets the URI of this resource

setVocabulary
:   OntResource::setVocabulary() in OntResource.php

    Sets the reference to the assoiated vocabulary

size
:   MemModel::size() in MemModel.php

    Number of triples in the MemModel

size
:   InfModelB::size() in InfModelB.php

    Number of all inferable triples in the Model.

size
:   ResContainer::size() in ResContainer.php

    Returns the number values in the container.

size
:   ResModel::size() in ResModel.php

    Number of statements in the MemModel

size
:   DbModel::size() in DbModel.php

    Return the number of statements in this DbModel

SOME\_VALUES\_FROM
:   OWLVocabulary::SOME\_VALUES\_FROM() in OWLVocabulary.php

Statement
:   Statement::Statement() in Statement.php

    The parameters to constructor are instances of classes and not just strings

Statement
:   Statement in Statement.php

    An RDF statement.

StatementIterator
:   StatementIterator in StatementIterator.php

    Iterator for traversing models.

StatementIterator
:   StatementIterator::StatementIterator() in StatementIterator.php

    Constructor

subject
:   Statement::subject() in Statement.php

    Alias for getSubject()

subtract
:   DbModel::subtract() in DbModel.php

    Return a new MemModel that is the subtraction of another model from this DbModel.

subtract
:   MemModel::subtract() in MemModel.php

    Returns a new MemModel that is the subtraction of another model from this MemModel.

subtract
:   ResModel::subtract() in ResModel.php

    Returns a new model that is the subtraction of another model from this model.

SUB\_CLASS\_OF
:   RdfsVocabulary::SUB\_CLASS\_OF() in RdfsVocabulary.php

    Answer the predicate that denotes that one class is a sub-class of another.

SUB\_CLASS\_OF
:   OWLVocabulary::SUB\_CLASS\_OF() in OWLVocabulary.php

    Answer the predicate that denotes that one class is a sub-class of another.

SUB\_PROPERTY\_OF
:   RdfsVocabulary::SUB\_PROPERTY\_OF() in RdfsVocabulary.php

    Answer the predicate that denotes that one property is a sub-property of another.

SUB\_PROPERTY\_OF
:   OWLVocabulary::SUB\_PROPERTY\_OF() in OWLVocabulary.php

    Answer the predicate that denotes that one property is a sub-property of another.

SYMMETRIC\_PROPERTY
:   OWLVocabulary::SYMMETRIC\_PROPERTY() in OWLVocabulary.php

StatementIterator.php
:   StatementIterator.php in StatementIterator.php

t

top

$Tokens
:   N3Parser::$Tokens in N3Parser.php

TriXParser.php
:   TriXParser.php in TriXParser.php

TriXSerializer.php
:   TriXSerializer.php in TriXSerializer.php

THING
:   OWLVocabulary::THING() in OWLVocabulary.php

toString
:   BlankNode::toString() in Blanknode.php

    Dumps bNode.

toString
:   Quad::toString() in Quad.php

    Return a human-readable (sort of) string "graphname { s p o . }" describing the quad.

toString
:   Resource::toString() in Resource.php

    Dumps resource.

toString
:   DbModel::toString() in DbModel.php

    Short dump of the DbModel.

toString
:   Statement::toString() in Statement.php

    Dumps the triple.

toString
:   Object::toString() in Object.php

    Serializes a object into a string

toString
:   InfModelB::toString() in InfModelB.php

    Short Dump of the InfModelB.

toString
:   Literal::toString() in Literal.php

    Dumps literal.

toString
:   InfModelF::toString() in InfModelF.php

    Short Dump of the InfModelF.

toString
:   MemModel::toString() in MemModel.php

    Short Dump of the MemModel.

toStringIncludingTriples
:   DbModel::toStringIncludingTriples() in DbModel.php

    Dump of the DbModel including all triples.

toStringIncludingTriples
:   MemModel::toStringIncludingTriples() in MemModel.php

    Dumps of the MemModel including all triples.

toStringIncludingTriples
:   InfModelB::toStringIncludingTriples() in InfModelB.php

    Dumps of the InfModelB including ALL inferable triples.

toStringObject
:   Statement::toStringObject() in Statement.php

    Reurns a toString() serialization of the statements's object.

toStringPredicate
:   Statement::toStringPredicate() in Statement.php

    Returns a toString() serialization of the statements's predicate.

toStringSubject
:   Statement::toStringSubject() in Statement.php

    Returns a toString() serialization of the statements's subject.

TRANSITIVE\_PROPERTY
:   OWLVocabulary::TRANSITIVE\_PROPERTY() in OWLVocabulary.php

TriXParser
:   TriXParser::TriXParser() in TriXParser.php

    Constructor Needs a reference to a graphSet

TriXParser
:   TriXParser in TriXParser.php

    Temporary implementation of a TriX-Parser (Usable only with PHP > V5) Currently, it doesn't support any namespaces and has problems with typed literals.

TriXSerializer
:   TriXSerializer in TriXSerializer.php

    Temporary implementation of a TriX-Serializer

TriXSerializer
:   TriXSerializer::TriXSerializer() in TriXSerializer.php

    Constructor Needs a reference to a graphSet

TYPE
:   OWLVocabulary::TYPE() in OWLVocabulary.php

    Answer the predicate that denotes the rdf:type property.

TYPE
:   RdfsVocabulary::TYPE() in RdfsVocabulary.php

    Answer the predicate that denotes the rdf:type property.

u

top

$use\_attributes
:   RdfSerializer::$use\_attributes in RdfSerializer.php

$use\_entities
:   RdfSerializer::$use\_entities in RdfSerializer.php

$use\_xml\_declaration
:   RdfSerializer::$use\_xml\_declaration in RdfSerializer.php

UNION\_OF
:   OWLVocabulary::UNION\_OF() in OWLVocabulary.php

unite
:   MemModel::unite() in MemModel.php

    Returns a new MemModel that is the set-union of the MemModel with another model.

unite
:   DbModel::unite() in DbModel.php

    Return a new MemModel that is the set-union the model with another model.

unite
:   ResModel::unite() in ResModel.php

    Returns a new Model that is the set-union of the model with another model.

uparse
:   N3Parser::uparse() in N3Parser.php

    This parses a N3 string and calls func($subject, $predicate, $object) with each trioke

v

top

valid
:   IteratorAllGraphsDb::valid() in IteratorAllGraphsDb.php

    Says if there are additional items left in the list

valid
:   ResIterator::valid() in ResIterator.php

    Says if there are additional items left in the list

valid
:   IteratorFindQuadsMem::valid() in IteratorFindQuadsMem.php

    Says if there are additional items left in the list

valid
:   IterFind::valid() in IterFind.php

    Says if there are additional items left in the list

valid
:   IteratorAllGraphsMem::valid() in IteratorAllGraphsMem.php

    Says if there are additional items left in the list

valid
:   IteratorFindQuadsDb::valid() in IteratorFindQuadsDb.php

    Says if there are additional items left in the list

VERSION\_INFO
:   OWLVocabulary::VERSION\_INFO() in OWLVocabulary.php

visualize
:   Model::visualize() in Model.php

    Alias for RDFUtil::visualiseGraph(&$model, $format, $short\_prefix)

visualizeGraph
:   RDFUtil::visualizeGraph() in RdfUtil.php

    Generates a dot-file for drawing graphical output with the

w

top

writeAsHtml
:   DbModel::writeAsHtml() in DbModel.php

    Write the RDF serialization of the \_DbModel as HTML.

writeAsHtml
:   MemModel::writeAsHtml() in MemModel.php

    Writes the RDF serialization of the MemModel as HTML.

writeAsHtml
:   InfModelB::writeAsHtml() in InfModelB.php

    Writes the RDF serialization of the Model including ALL inferable triples as HTML.

writeAsHtmlTable
:   MemModel::writeAsHtmlTable() in MemModel.php

    Writes the RDF serialization of the MemModel as HTML table.

writeAsHTMLTable
:   ResModel::writeAsHTMLTable() in ResModel.php

    Writes the RDF serialization of the MemModel as HTML table.

writeAsHtmlTable
:   InfModelB::writeAsHtmlTable() in InfModelB.php

    Writes the RDF serialization of the Model including ALL inferable triples as HTML table.

writeAsHtmlTable
:   DbModel::writeAsHtmlTable() in DbModel.php

    Write the RDF serialization of the DbModel as HTML table.

writeHTMLTable
:   RDFUtil::writeHTMLTable() in RdfUtil.php

    Prints a MemModel as HTML table.

writeRdfToString
:   ResModel::writeRdfToString() in ResModel.php

    Writes the RDF serialization of the MemModel as HTML table.

writeRdfToString
:   InfModelB::writeRdfToString() in InfModelB.php

    Writes the RDF serialization of the Model including ALL inferable triples.

writeRdfToString
:   DbModel::writeRdfToString() in DbModel.php

    Write the RDF serialization of the DbModel to string

writeRdfToString
:   MemModel::writeRdfToString() in MemModel.php

    Writes the RDF serialization of the MemModel as HTML table.

\_

top

\_generateRDF
:   GRDDLParser::\_generateRDF() in GRDDLParser.php

\_is\_forbidden\_rdf\_property\_attribute
:   RdfParser::\_is\_forbidden\_rdf\_property\_attribute() in RdfParser.php

\_parse\_uri
:   RdfParser::\_parse\_uri() in RdfParser.php

\_report\_error
:   RdfParser::\_report\_error() in RdfParser.php

a
b
c
d
e
f
g
h
i
k
l
m
n
o
p
q
r
s
t
u
v
w
\_
